# Slit2/Robo1 signaling inhibits small‐cell lung cancer by targeting β‐catenin signaling in tumor cells and macrophages

**DOI:** 10.1002/1878-0261.13289

**Published:** 2023-01-10

**Authors:** Dinesh K. Ahirwar, Bo Peng, Manish Charan, Swati Misri, Sanjay Mishra, Kirti Kaul, Salha Sassi, Venkat Sundar Gadepalli, Jalal Siddiqui, Wayne O. Miles, Ramesh K. Ganju

**Affiliations:** ^1^ Department of Pathology, College of Medicine The Ohio State University Columbus OH USA; ^2^ Comprehensive Cancer Center The Ohio State University Columbus OH USA; ^3^ Department of Bioscience & Bioengineering Indian Institute of Technology Jodhpur India; ^4^ Biomedical Informatics The Ohio State University Columbus OH USA; ^5^ Department of Cancer Biology and Genetics The Ohio State University Columbus OH USA

**Keywords:** MDSCs, Robo1, Slit2, small‐cell lung cancer, TAMs

## Abstract

Small‐cell lung cancer (SCLC) is an aggressive neuroendocrine subtype of lung cancer with poor patient prognosis. However, the mechanisms that regulate SCLC progression and metastasis remain undefined. Here, we show that the expression of the slit guidance ligand 2 (*SLIT2*) tumor suppressor gene is reduced in SCLC tumors relative to adjacent normal tissue. In addition, the expression of the SLIT2 receptor, roundabout guidance receptor 1 (*ROBO1*), is upregulated. We find a positive association between *SLIT2* expression and the Yes1 associated transcriptional regulator (*YAP1*)‐expressing SCLC subtype (SCLC‐Y), which shows a better prognosis. Using genetically engineered SCLC cells, adenovirus gene therapy, and preclinical xenograft models, we show that *SLIT2* overexpression or the deletion of *ROBO1* restricts tumor growth *in vitro* and *in vivo*. Mechanistic studies revealed significant inhibition of myeloid‐derived suppressor cells (MDSCs) and M2‐like tumor‐associated macrophages (TAMs) in the SCLC tumors. In addition, SLIT2 enhances M1‐like and phagocytic macrophages. Molecular analysis showed that *ROBO1* knockout or *SLIT2* overexpression suppresses the transforming growth factor beta 1 (TGF‐β1)/β‐catenin signaling pathway in both tumor cells and macrophages. Overall, we find that SLIT2 and ROBO1 have contrasting effects on SCLC tumors. SLIT2 suppresses, whereas ROBO1 promotes, SCLC growth by regulating the Tgf‐β1/glycogen synthase kinase‐3 beta (GSK3)/β‐catenin signaling pathway in tumor cells and TAMs. These studies indicate that SLIT2 could be used as a novel therapeutic agent against aggressive SCLC.

## Graphical abstract

In patients with small‐cell lung cancer (SCLC), the expression of SLIT2 is greatly reduced whereas the SLIT2 receptor, roundabout guidance receptor 1 (ROBO1), is significantly elevated. *SLIT2* overexpression or the deletion of *ROBO1* restricts tumor growth *in vitro* and *in vivo*. In addition, SLIT2 treatment enhances M1‐like phagocytic macrophages and reduces M2‐like tumor‐associated macrophages (TAMs) in SCLC tumors. Molecular analysis showed that *SLIT2* overexpression or *ROBO1* deletion suppresses the transforming growth factor‐beta 1 (TGF‐β1)/β‐catenin signaling pathway in both tumor cells and macrophages.

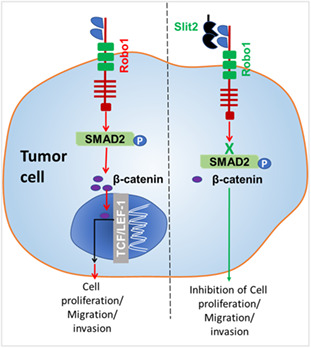

**Abbreviations**
Ad‐Null: adenovirus expressing vector control
Ad‐Slit2: adenovirus expressing Slit2
ASCL1: achaete‐scute family BHLH transcription factor 1
BMDMs: bone marrow‐derived macrophages
CD206: cluster of differentiation 206
CD31: cluster of differentiation 31
CD45: cluster of differentiation 45
DAPI: 4′,6‐diamidino‐2‐phenylindole
EMT: epithelial‐to‐mesenchymal transition
GSK‐3: glycogen synthase kinase‐3
MDSCs: myeloid‐derived suppressor cells
MOI: multiplicity of infection
NEUROD1: neuronal differentiation 1
PFU: plaque‐forming unit
POU2F3: POU class 2 homobox 3
ROBO1: roundabout guidance receptor 1
SCLC: small‐cell lung cancer
SLIT2: slit guidance ligand 2
TAMs: tumor associate macrophages
TBS: tris buffer saline
TGF‐β1: transforming growth factor beta 1
TME: tumor microenvironment
YAP1: Yes1 associated transcriptional regulator

## 1 Introduction

Lung cancer is the second most common cancer type and remains the leading cause of cancer‐related deaths worldwide [1]. Among different types of lung cancers, small‐cell lung cancer (SCLC) is the most aggressive subtype and is defined by neuroendocrine features, rapid growth, and high death rate [2, 3]. The major cause of death is metastasis, and the 5‐year survival rate is only 3% for patients with metastatic SCLC [4]. SCLC is difficult to treat, and several acquired drug resistance mechanisms have been identified. These issues have resulted in the treatment regime for SCLC to remain unchanged for the last four decades [5]. Recently approved by FDA, immune checkpoint inhibitors provide only marginal benefits for SCLC patients [5, 6]. There is therefore a significant need to identify new molecular targets and therapeutic strategies for SCLC treatment.

Genome‐wide screening studies have identified several tumor suppressor genes in SCLC [7]. An integrated genomic analysis identified the Slit2 gene as a top driver gene, along with the biallelic inactivation of Tp53 and Rb1 genes in SCLC patients [7]. Other studies have shown that the Slit2 gene is suppressed in SCLC patients via several mechanisms including hypermethylation, loss of heterozygosity (LOH), and inactivating mutations [8, 9]. However, the role of Slit2 in cancer is highly context‐dependent. Several studies reported tumor‐suppressive functions of Slit2 in breast, colorectal, thyroid, and cervical cancers [10, 11, 12, 13, 14, 15, 16]. In the contrast, Slit2 has been shown to promote tumorigenesis in nasopharyngeal, skin, and intestinal cancers [17, 18, 19]. Slit2 is a large and secreted glycoprotein that functions to regulate axonal guidance, angiogenesis, and organ development [20, 21, 22, 23, 24]. The Slit2 protein binds to and activates the roundabout 1 (Robo1) receptor [25, 26, 27, 28]. A comprehensive genomic study has identified Robo1 gene mutations in SCLC patients [29]. Genetic and immunological inhibition of Robo1 has established its oncogenic role in multiple myeloma and prostate cancer *in vitro* and *in vivo* [30, 31, 32]. Genetic suppression of Slit2 or overexpression of Robo1 in hepatocellular carcinoma promotes tumor growth and metastasis [33]. However, the functional roles of Slit2/Robo1 signaling in SCLC pathogenesis and the therapeutic potential of targeting Slit2 in SCLC have not been evaluated.

Here, we show that both overexpression of Slit2 and deletion of Robo1 independently inhibit SCLC cells tumorigenic properties *in vitro* and *in vivo*. We found that these manipulations significantly inhibited proliferative and oncogenic signaling pathways downstream of Robo1. Mechanistic studies showed that the Slit2/Robo1 signaling prevents the polarization of pro‐tumor M2‐like macrophages in the tumor microenvironment (TME) by targeting Tgf‐β1 signaling. Furthermore, Slit2 treatment increased the number of M1‐like phagocytic macrophages in SCLC tumors. By profiling human SCLC samples, we found widespread reductions in Slit2 levels in SCLC and that elevated Slit2 levels were found in SCLC subtypes with longer survival than other subtypes. In agreement, we found that Robo1 expression was increased in SCLC patients. Overall, we show that Slit2/Robo1 signaling is tumor suppressive in SCLC, suggesting that Slit2 may represent an ideal substrate for the development of an immune‐based therapeutic agent.

## 2 Materials and methods

### 2.1 Animal studies

All experiments were approved by the Institutional Animal Care and Use Committee of the Ohio State University and performed as per our approved animal protocol (2007A0233‐R4). Animals were housed with regards to food, water, and cages as per University Laboratory Animal Resources guidelines. Animal experiments were performed with the Nude male mice (Strain Foxn1 nu) obtained from targeted validation shared resources (TVSR) at the Ohio State University (OSU), USA. The Nude male mice of 6 weeks were used for subcutaneous implantation of SBC5 xenograft tumor growth studies. For subcutaneous tumor implantation, a total of 1 × 10^6^ SBC5 cells in 100 μL of sterile saline were injected into the right flank of nude mice. At tumor palpation, mice were randomly divided into two groups and treated with Adenovirus expressing human Slit2 (Ad‐Slit2) or empty vector control (Ad‐Null) in 100 μL of PBS (1 × 10^7^ PFU, weekly, i.p.) for 4 weeks. Tumors were measured weekly using a digital caliper and tumor volume was calculated.

### 2.2 Cell lines and culture

SBC5 cell line was a kind gift from Saburo Sone and Seiji Yano (University of Tokushima School of Medicine, Japan). SBC5 wild type, SBC5‐Robo1 knockout, or SBC5‐Vector control cells were cultured in DMEM (Lonza, Durham, NC, USA) supplemented with 10% FBS (Sigma‐Aldrich, St. Louis, MO, USA) and 1% penicillin/streptomycin (Lonza) at 5% CO_2_. Cells were transduced with Ad‐Slit2 or Ad‐Null at MOI 100.

### 2.3 Wound healing assay

The cells were seeded in six‐well plates to achieve 90% confluency the next day. The cells were serum‐starved for 4 h. A uniform wound was created along the central axis of the well using a 1 mL pipette tip and the wells were washed 3× with serum‐free media. The cells were incubated at 37 °C and the images of the wounds were taken at different time points. The imaging was performed using 20× objective of EVOS imaging system (AMEX 1000, ThermoFisher, Waltham, MA, USA).

### 2.4 Cell proliferation assay

Cell viability or proliferation was measured by PrestoBlue cell viability assay according to the manufacturer's instructions (Molecular Probes, Waltham, MA, USA). Briefly, cells were seeded in a 96‐well plate for 12 h at a density of 5000 cells per well and incubated for 0–72 h. Six replicates were used for each group. Results were expressed as the mean percentage of cell proliferation.

### 2.5 Flow cytometry analysis

The efficiency of ROBO1 knockout was detected by flow cytometry. 1 × 10^6^ cells were suspended in 100 μL of 5% BSA PBS and were incubated with the anti‐ROBO1 antibody on ice for 30–60 min, and rinsed in PBS, followed by incubating with Alexa Fluor 488‐conjugated secondary antibody in the dark for 30 min. After washing, cellular fluorescence intensity was recorded using flow cytometry.

#### 2.5.1 Tumor flow cytometry

Single‐cell suspension from tumors was analyzed by flow cytometry as described earlier [15]. Briefly, cells were incubated with Fc receptor blocker followed by staining with anti‐CD45‐ Brilliant Violate 421, anti‐F4/80 PerCP/Cy5.5, anti‐CD11b APC/Cy7, anti‐CD206 PE/Cy7, anti‐CCR7 Brilliant Violate 785, and anti‐Gr1 FITC (BioLegend, San Diego, CA, USA). For staining intracellular targets, cells were fixed and permeabilized for 30 min at room temperature (Fixation/Permeabilization Diluent, eBioscience, Waltham, MA, USA). Cells were stained with anti‐EpCAM‐APC (BioLegend). A total of 10 × 10^6^ cells were recorded on a flow cytometer. All the data was recorded on FACS Fortessa (BD Biosciences, Waltham, MA, USA) and analyzed using flowjo software (FlowJo LLC, Ashland, OR, USA).

### 2.6 Colony formation assay

Cells were seeded into the 6‐well plates at 500–1000 cells per well. The formed cell colonies were fixed with 4% paraformaldehyde (Fisher Scientific) at room temperature for 20 min and stained using Hema 3 staining kit as per the manufacturer's instructions (Fisher Scientific). The number of colonies and the colony area was calculated using imagej (NIH, Bethesda, MD, USA).

### 2.7 Cell migration and invasion assay

For transwell migration assay, 1 × 10^5^ cells in 0.1 mL serum‐free conditional medium were seeded on the upper compartment of the chamber (6.5 mm diameter filters, 8 μm pore size, Corning, Glandale, AZ, USA), and 0.6 mL of medium with 10% FBS was added to the lower compartment. After incubation for 6–10 h, the cells on the lower surface of the filter were fixed and stained. Migrated cells on the bottom side of the membranes were counted. For transwell invasion assay, Boyden chamber pre‐coated with matrigel (20 μL per well, 1.2 mg·mL^−1^) at 37 °C for 2 h. A similar protocol was followed as the abovementioned migration assay.

### 2.8 Immunofluorescence

The standard immunofluorescence procedure was followed. Briefly, cells were fixed with 4% paraformaldehyde at room temperature for 20 min. Cells were washed with PBS, blocked with 5% goat serum in Tris‐buffered saline with Tween 20 (TBST) buffer for 60 min, and incubated with anti‐β‐catenin antibody (1 : 100 dilution, clone D10A8, Cell Signaling Technologies, Danvers, MA, USA) overnight at 4 °C followed by incubation with anti‐rabbit secondary antibody conjugated with Alexa Flour 568 (1 : 500 dilution, Fisher Scientific) for 60 min. Cells were washed with TBST and mounted using vectashield mounting medium with DAPI and examined under Olympus FV1000 Filter confocal microscope at 400× magnification (Olympus, Pittsburgh, PA, USA).

### 2.9 Immunohistochemistry

Tumor sections (4 μm) were analyzed using standard IHC techniques as per the manufacturer's recommendations (Vector Laboratories, Burlingame, CA, USA). Briefly, the paraffin‐embedded section on the slide was processed through three changes of xylene, followed by hydrating the tissue in a gradient of alcohol (100%, 95%, 90%, 80%, 70%, and 50% reagent alcohol with distilled water). The hydrated tissue was boiled in a pressure cooker with sodium citrate buffer at acidic pH for 20 min for antigen retrieval. The tissues were rinsed with 1X Tris buffer saline (TBS) and treated with hydrogen peroxide for 10 min at room temperature. The tissues were washed 2× with 1× TBS and incubated with anti‐mouse antibodies against CD31 (Cell Signaling Technologies, 1 : 200), or β‐catenin (Cell Signaling Technologies, 1 : 200) at 4 °C overnight, followed by incubating with anti‐rabbit ImmPRESS polymer reagent tagged with horseradish peroxidase or alkaline phosphatase (Vector Laboratories). Enzyme‐specific chromogen color development was performed using ImmPACT‐DAB or ImmPACT‐red to detect the bound primary antibodies. The cell nucleus was counterstained with hematoxylin (Vector Laboratories). The images were acquired using a brightfield microscope (Keyence, Itasca, IL, USA) at 200× magnification.

### 2.10 RNA sequencing and data analysis

RNA sequencing and data analysis were performed using the previously published sample preparation techniques and data analysis pipelines [10]. Briefly, total RNA was collected from SBC5‐scr or SBC5‐Robo1 knockout cells using RNeasy Plus Mini Kit (Qiagen, Germantown, MD, USA). The purity and integrity of RNA were analyzed on a Bioanalyzer 2100 (Agilent, Santa Clara, CA, USA) and Qubit Fluorimeter (Invitrogen, Waltham, MA, USA). RNA sequencing (RNA‐seq) was performed by genomic core facilities at the Ohio State University (Columbus, OH). The RNA integrity number values were > 7 and the RNA concentration was > 100 ng·μL^−1^ for all samples. mRNA‐sequencing libraries were generated with NEBNext^®^ Ultra™ II Directional RNA Library Prep Kit for Illumina (NEB) and NEBNext Poly(A) mRNA Magnetic Isolation Module (NEB) with an input amount of 200 ng total RNA per sample. Libraries were pooled and sequenced on an Illumina NovaSeq SP flow cell in paired‐end 150 bp format (Illumina, San Diego, CA, USA) to a read yield between 35 and 40 million reads.

The Analysis was performed using an in‐house pipeline BISR‐RNA‐seq [34]. Gene‐wise counts were created with feature counts from the subread package v1.5.1 [35] for the genes annotated through human GRCh38/hg38, counting the primary alignment in the case of multi‐mapped reads. Voom was utilized to normalize raw counts and limma was used for differential expression analysis [36, 37]. Genes were tested whether at least 66% of the samples had an expression of 2 counts per million (CPM). Two comparisons were made to assess differential expression between groups: ps_scr versus ps_Robo1‐knockout. Significant genes had FDR < 0.05 and logFC > 1 or ←1. Ingenuity Pathway (IP) analysis was performed using significant genes.

### 2.11 Database mining

Files containing RNA sequencing reads were adaptor and quality‐trimmed using trimgalore 0.6.6. bowtie2 (version 2.4.4) was used to remove contaminating reads from ribosomal RNA and transfer RNA [38, 39, 40]. The tximport function from tximport bioconductor package [41] alongside a TxDB database [42] created from GENCODE Human Release 31 annotation GTF file was used to obtain gene expression counts. Differential expression analysis was performed on the gene counts using DESeq2 [43].

### 2.12 Statistical analysis

Statistical analyses were performed using graphpad prism 6.0 Software (GraphPad Software, San Diego, CA, USA). Data were expressed as mean ± SD. Means of all data were compared by unpaired *t*‐test or one‐way ANOVA followed by *post hoc* Dunnett's test. In each case, the *P*‐value of < 0.05 was considered statistically significant.

## 3 Results

### 3.1 Slit2 and Robo1 expression patterns are altered in SCLC

**Fig. 1:**
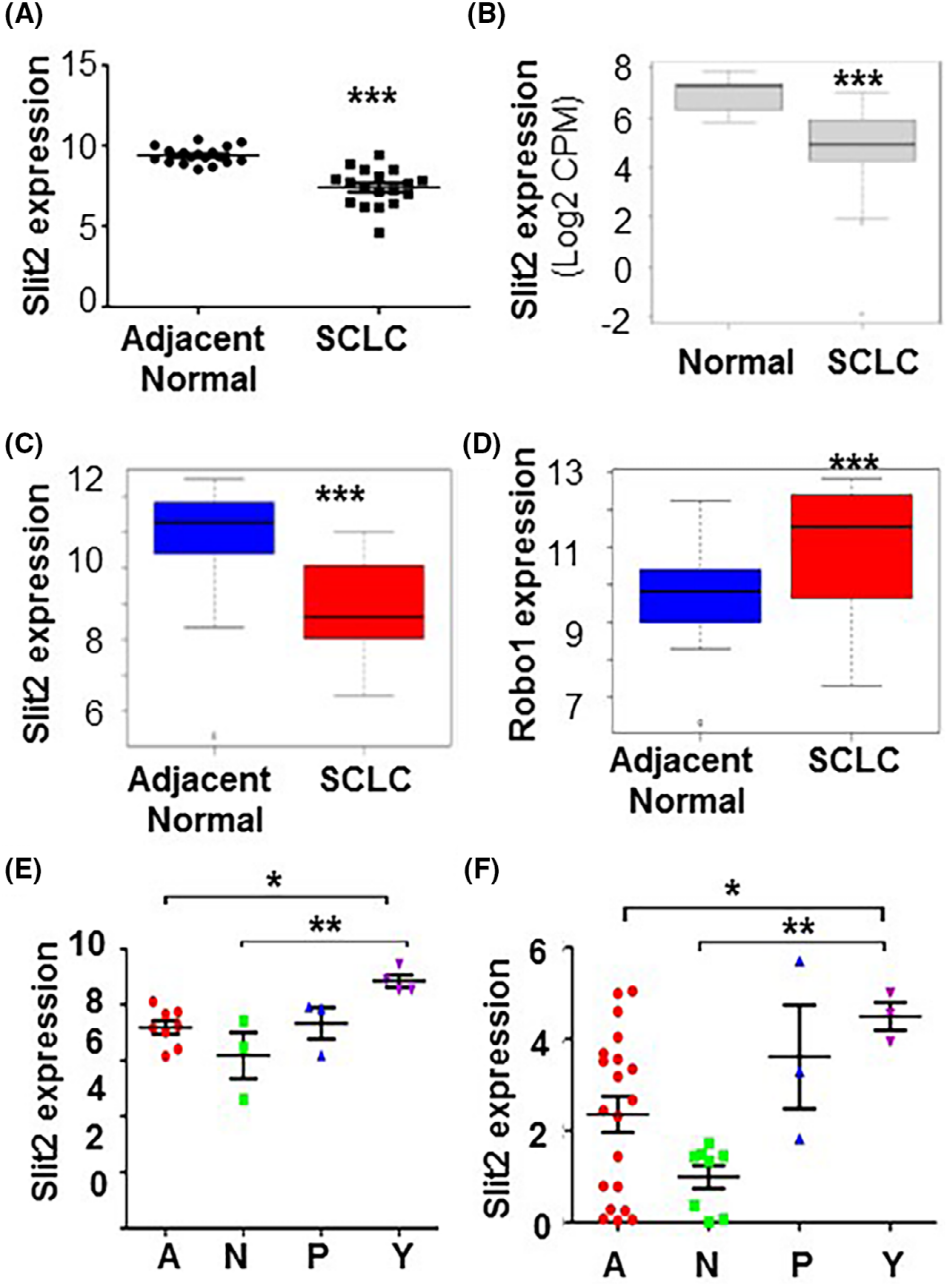
Expression of Slit2 and Robo1 in SCLC patients. (A–D) Slit2 expression was analyzed in human SCLC tumors and adjacent normal tissues using publicly available dataset containing (A) 18 samples of SCLC and matched adjacent normal (dataset GEO149507), (B) 7 samples of normal lungs and 79 SCLC (dataset GSE60052), and (C) 24 samples of SCLC and matched adjacent normal in EGAS000010000334 dataset. (D) Analysis of Robo1 expression using EGAS000010000334 dataset. (E) The SCLC from GEO149507 were analyzed for Slit2 expression among different subtypes of SCLC [SCLC‐A (a), SCLC‐N (N), SCLC‐P (P) and SCLC‐Y (Y)]. (F) Analysis of Slit2 expression in SCLC cell lines of different subtypes using publicly available dataset SCLC CellMiner. All the error bars represent SD. *T*‐test was used to compare two groups (A–D) and one‐way ANOVA to compare multiple groups (E, F). * is statistical *P* value < 0.05, ** < 0.01, and *** < 0.001.

To explore the significance of Slit2 in SCLC, we analyzed the expression of Slit2 in publicly available datasets. We first evaluated the expression of Slit2 in SCLC patients (*n* = 18) and observed that SCLC tumors have reduced levels of Slit2 mRNA compared with adjacent normal tissue (Fig. 1A) [44]. We next analyzed two independent SCLC patient cohorts to control for patient and tumor heterogeneity which, respectively, contained seven normal tissues and 79 SCLC tissues [45], and 24 adjacent normal and SCLC tissues [46], and found diminished Slit2 levels in SCLC tumors (Fig. 1B,C). In contrast, Robo1 levels were increased in SCLC patient samples compared with adjacent normal (Fig. 1D). These findings showed that Slit2/Robo1 levels are altered in SCLC. To test whether Slit2/Robo1 was SCLC subtype‐specific, we sub‐divided tumors based on the expression of transcription factors, ASCLC1 (SCLC‐subtype A), POU2F3 (SCLC‐subtype P), NEUROD1 (SCLC‐subtype N), and YAP1 (SCLC‐subtype Y) (Table S1) [47]. From this analysis, Slit2 expression in SCLC tumors and cell lines is higher in the SCLC‐Y subtype (Fig. 1E,F) [48]. Recent studies have shown that SCLC‐Y correlates with longer patient survival compared with other subtypes [49]. These results suggest that Slit2 expression is reduced in SCLC tumors, however, this is subtype dependent, with Slit2 levels highest in SCLC patients from the SCLC‐Y subtype.

### 3.2 Slit2 overexpression suppresses SCLC tumorigenic phenotypes *in vitro*

**Fig. 2:**
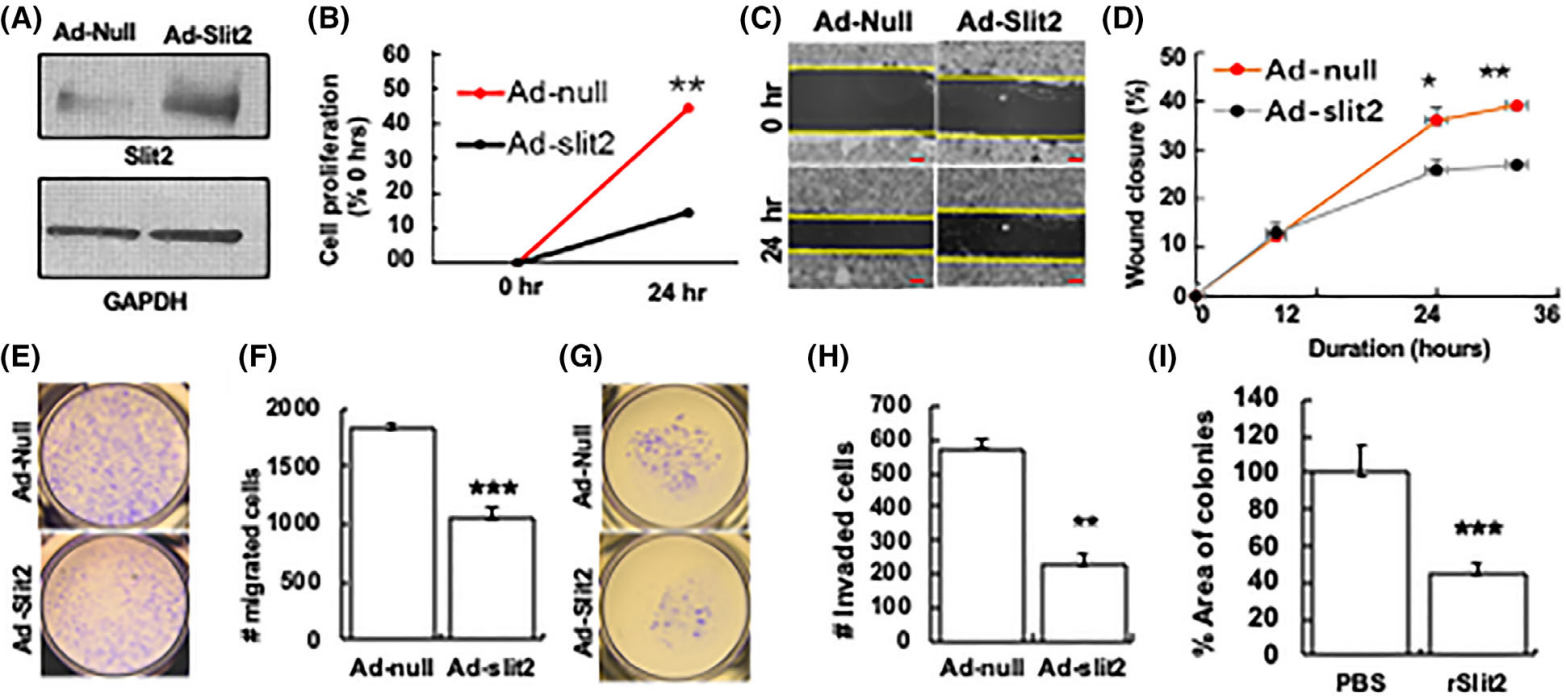
Slit2 inhibits tumorigenic properties of SCLC cells. (A) SBC5 cells were transduced with adenovirus expressing Slit2 (ad‐Slit2) of vector control (ad‐null) and the cell lysates were analyzed for Slit2 levels by western blot. GAPDH was used as a loading control. SBC‐5 cells transduced with ad‐null or ad‐Slit2 were subjected to (B) PrestoBlue cell viability assay, (C, D) wound healing assay (scale bar is 100 μm), (E, F) cell migration assay, and (G, H) Matrigel invasion assay. (I) SBC5 cells were treated with PBS or rSlit2 and subjected to colony formation assay. The area of colonies was calculated using imagej software (NIH). All quantitative data were presented as mean ± SD of three replicates from three independent experiments. *T*‐test was used for the comparison of two groups. *P < 0.05, **P < 0.01, ***P < 0.001 using student's *t*‐test.

As Slit2 expression is reduced in SCLC patients, we tested how changes in Slit2 levels affected SCLC phenotypes *in vitro*. For this, we transduced SBC‐5 cells with Adenovirus expressing Slit2 (Ad‐Slit2) or empty vector (Ad‐null) to generate Slit2 overexpressing SBC5 (SBC5‐Ad‐Slit2) or vector control (SBC5‐Ad‐Null) cells. Western blot analysis of these cells confirmed overexpression of Slit2 in SBC5‐Ad‐Slit2 compared with SBC5‐Ad‐Null cells (Fig. 2A). We next tested how Slit2 overexpression altered the tumorigenic properties of SCLC cells. Cell proliferation assays showed that Slit2 overexpression significantly inhibited the growth of SBC‐5 cells (Fig. 2B). To assess the role of Slit2 in the metastatic potential of cancer cells, scratch assays and transwell migration assays were used. Slit2 overexpression in SBC‐5 cells significantly reduced cell migration rates (Fig. 2C,D), the number of migrated cells (Fig. 2E,F), and the number of cells with Matrigel invasive properties (Fig. 2G,H). Similarly, we observed a reduced ability to form colonies in SBC5 cells treated with recombinant Slit2 (rSlit2) compared with PBS control (Fig. 2I). These findings strongly support the role of Slit2 having a tumor suppressor role in SCLC cells.

### 3.3 Genetic deletion of Robo1 suppresses SCLC phenotypes *in vitro*

**Fig. 3:**
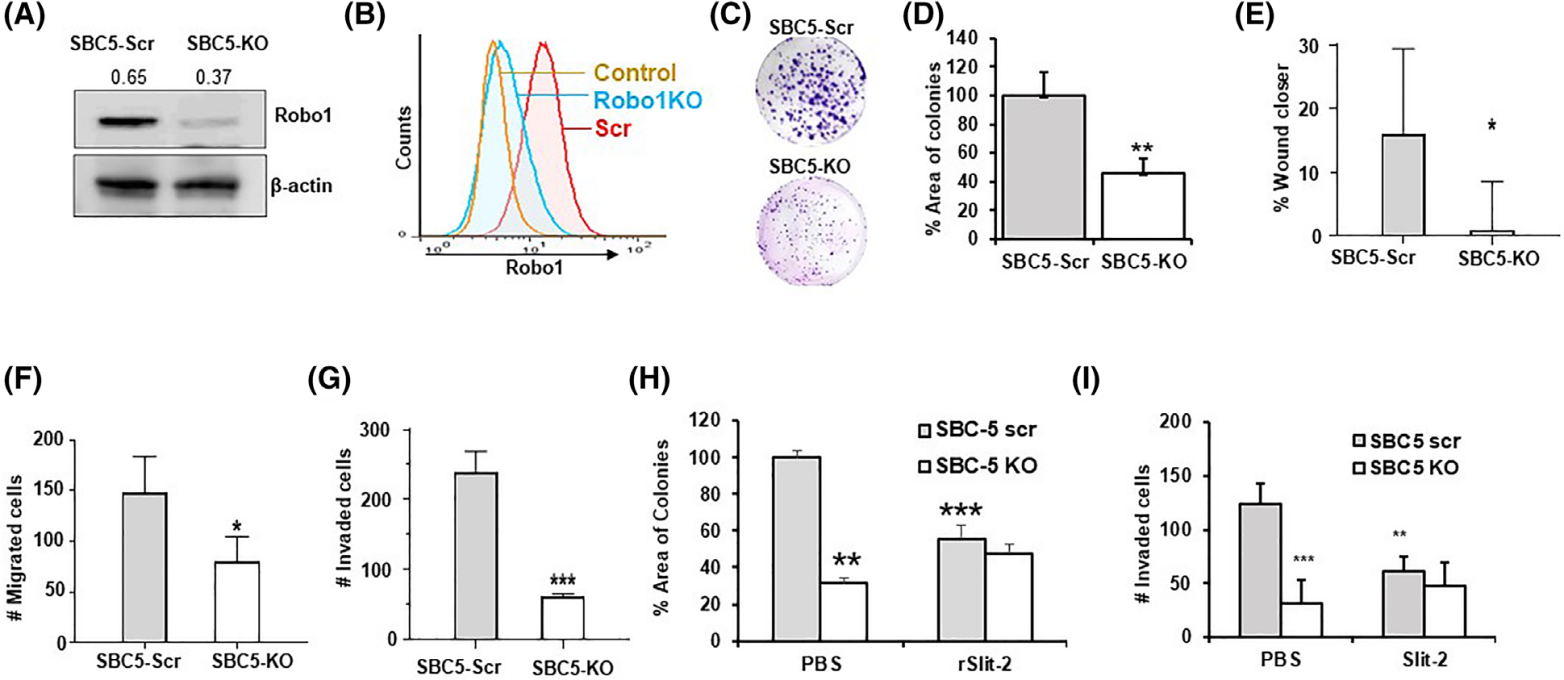
Robo1 knockout inhibits tumorigenic properties of SCLC cells. Robo1 knockout (SBC5‐KO) or scrambled control (SBC5‐Scr) SBC5 cells were generated and analyzed for Robo1 levels by (A) western blot and (B) flow cytometry. IgG was used as antibody isotype control (control). (C–G) SBC5‐Scr or SBC5‐KO cells were analyzed for (C) the potential of colony formation, (D) quantification of percent area of colonies from (C), (E) % wound closer by wound closer assay and (F) transwell migration assay, and (G) cell invasion was determined by cell matrigel coated transwell invasion assay. (H, I) SBC5‐Scr or SBC5‐KO cells were treated with PBS or Slit2 and analyzed for (H) percent area of cell colonies, and (I) number of cells invaded the matrigel layer. All quantitative data are presented as mean ± SD from three replicates of one experiment, and all experiments were repeated at least three times with similar results. *P < 0.05; **P < 0.01; ***P < 0.001 using student's *t* test for comparing two groups or one way ANOVA test for comparing multiple groups.

Slit2 has been shown to bind to Robo1 and trigger proliferation cascades [25, 27]. To explore the role of Slit2/Robo1 signaling in SCLC, we generated Robo1 knockout SBC‐5 cells (SBC5‐Robo1KO) or scrambled control (SBC5‐Scr) cell lines using CRISPR/Cas9 technology. The efficiency of Robo1 knockout was detected by western blot (Fig. 3A) and flow cytometry analysis (Fig. 3B). We then tested how Robo1 KO changed the tumorigenic properties of cells, and found significantly reduced colony formation (Fig. 3C,D), wound closure (Fig. 3E), cell migration (Fig. 3F), and cell invasion (Fig. 3G). As Robo1 directly interacts with Slit2, we tested the effect of rSlit2 treatment on the tumorigenic properties of control and Robo1 KO SCLC cells. Colony formation assays showed that control (PBS)‐treated SBC5‐Scr cells were able to form colonies at significantly higher levels compared with rSlit2‐treated SBC5‐Scr or SBC5‐Robo1KO or rSlit2‐treated SBC5‐Robo1KO cells (Fig. 3H). Similarly, the invasive property of PBS‐treated SBC5‐Scr was significantly higher compared with rSlit2‐treated SBC5‐Scr or SBC5‐Robo1KO or rSlit2‐treated SBC5‐Robo1KO cells (Fig. 3I). These results highlight the role of the Slit2/Robo1 pathway in regulating SCLC growth.

### 3.4 Slit2 inhibits while Robo1 promotes SCLC growth *in vivo*

**Fig. 4:**
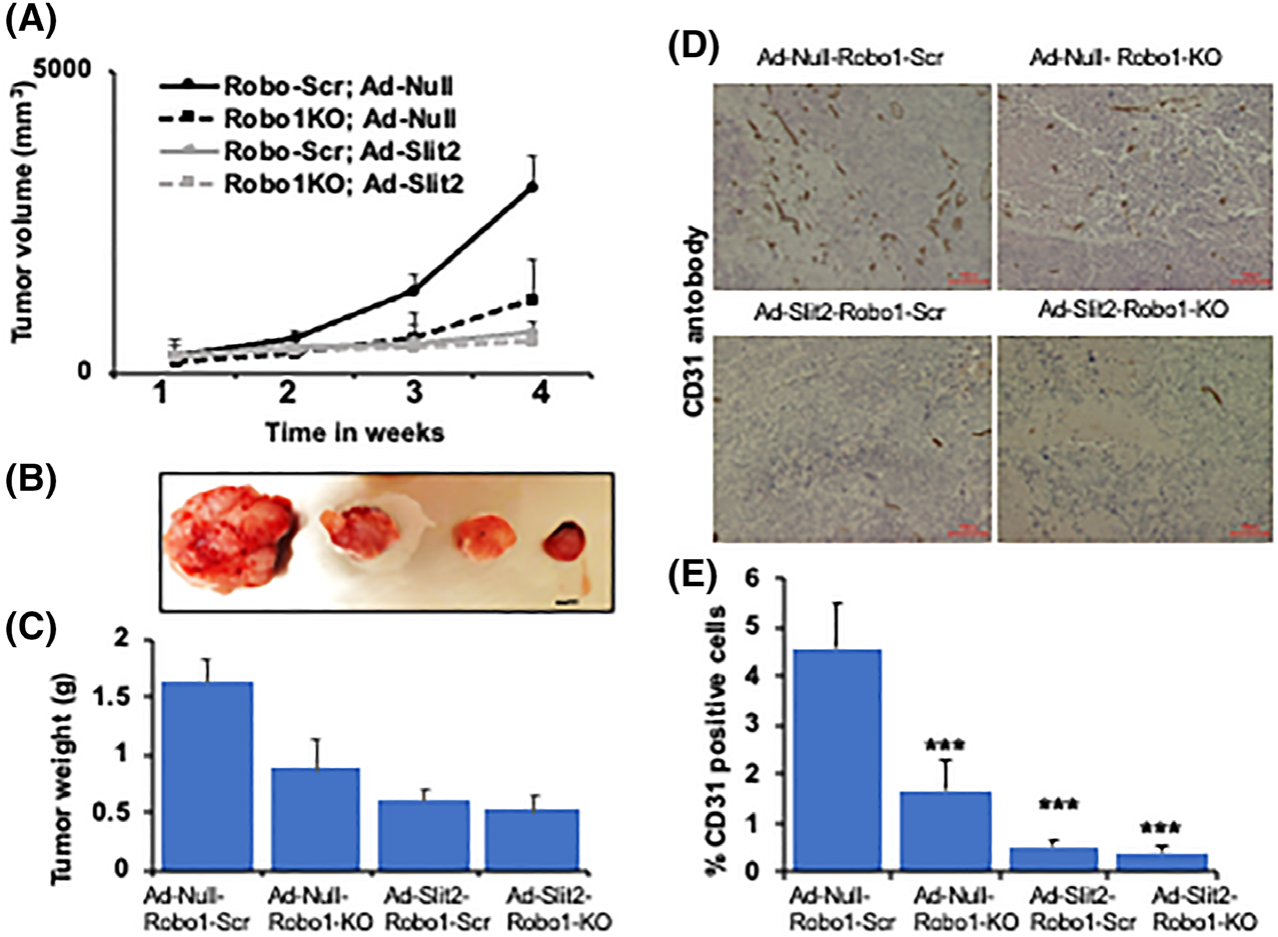
Slit2/Robo1 signaling inhibits SCLC growth *in vivo*. SBC5‐Scr or SBC5‐Robo1KO cells were injected into the nude mice subcutaneously. At tumor palpation, the mice were treated with adeno‐null or ad‐Slit2 weekly for 4 weeks. (A) Graph depicting tumor volume recorded at different time points. (B) Representative images of the harvested tumors (scale bar 2 mm). (C) Weight of harvested tumors. (D) Representative images of CD31 IHC analysis on harvested tumor tissues (scale bar 100 μm). (E) Quantification CD31 positive cells by imagej software (NIH). All quantitative data were presented as mean ± SD of three replicates from three independent experiments. ***P < 0.001 using one way ANOVA test.

Next, we analyzed the role of Slit2/Robo1 in SCLC *in vivo*. We implanted SBC5‐Scr or SBC5‐Robo1KO subcutaneously into the nude mice and treated tumor‐bearing mice with Ad‐Slit2 or Ad‐Null. Weekly analysis of tumor volume showed that the rate of SBC5‐Robo1KO tumor growth is significantly reduced compared with SBC5‐Scr (Fig. 4A). We also observed a reduced weight of tumors harvested from Robo1KO tumors (Fig. 4B,C). Similarly, Ad‐Slit2‐treated SBC5‐Scr tumors showed significantly reduced tumor growth and tumor weight compared with control SBC5‐Scr (Fig. 4A–C). Interestingly, the SBC5‐Robo1 KO tumors treated with Ad‐Slit2 also showed the highest inhibition of tumor growth (Fig. 4A–C). Exponential tumor growth is supported by the upregulated rate of angiogenesis. We also analyzed if reduced tumor growth observed in Robo1 knockout and Slit2‐treated SBC xenografts is linked to reduced angiogenesis. Analysis of angiogenesis marker CD31 showed a marked reduction in angiogenesis in Slit2‐treated or Robo1KO tumors (Fig. 4D,E). These results suggest that the inhibition of Robo1 and/or treatment with Slit2 inhibits SCLC growth *in vivo*.

### 3.5 Slit2 inhibits macrophage polarization in SCLC

**Fig. 5:**
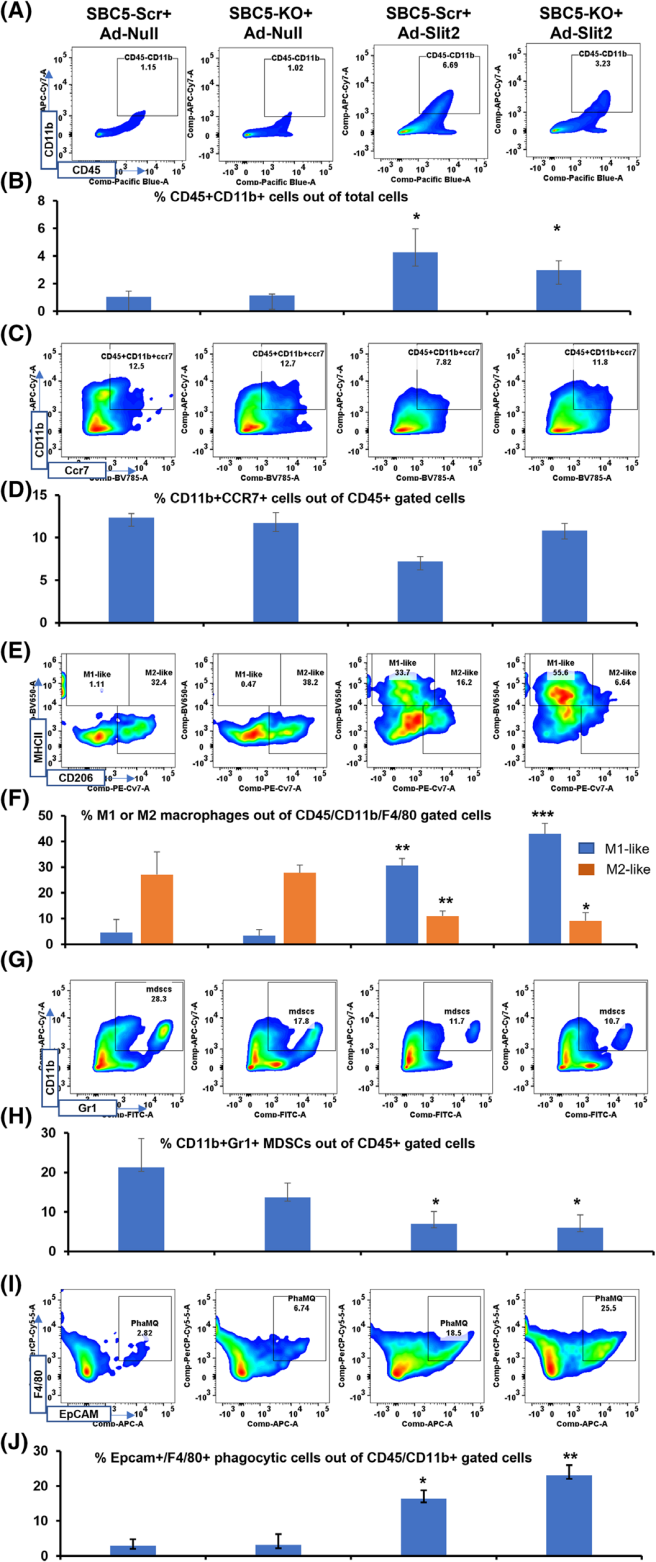
Slit2 suppresses pro‐tumor macrophages. SBC5‐Scr or SBC5‐Robo1KO (SBC5‐KO) xenografts treated with PBS or rSlit2 were analyzed for different subpopulations of myeloid cells by flow cytometry. (A) Representative flow cytometry plots of CD45^+^/CD11b^+^ total myeloid cells. (B) Quantification of total myeloid cells. (C) Representative flow cytometry plots of CD11b^+^/Ccr7^+^ myeloid cells. (D) Representative flow cytometry plots of MDSCs. (E) Quantification of CD11b^+^/Ccr7^+^ cells. (E) Representative cytometry plot showing detection of M1‐like and M2‐like macrophages. (F) Quantification of M1‐like and M2‐like macrophages. (G) Representative cytometry plot showing identification of MDSCs. (H) Quantification of MDSCs. (I) Cancer cell marker EpCAM positive macrophages were identified as phagocytic macrophages by flow cytometry. (J) Quantification of phagocytic macrophages. All quantitative data were presented as mean ± SD of three replicates from three independent experiments. *P < 0.05; **P < 0.01; ***P < 0.001 using one way ANOVA test.

As SBC5‐Robo1KO tumor growth was inhibited by rSlit2 treatment, we hypothesized that Slit2 may activate host immune cells to restrict tumor growth. As Nude mice lack T cells, we focused our analysis on the sub‐populations of innate cells known to be present in the tumors. Flow cytometry analysis of tumor samples showed that Slit2 treatment significantly enhanced tumor infiltration of CD45^+^/CD11b^+^ myeloid cells, in contrast to Robo1KO (Fig. 5A,B). Multiple studies have defined different subpopulations of myeloid cells in the lung TME, including CD11b^+^/Gr1^+^ myeloid‐derived suppressor cells (MDSCs), CD11b^+^ CCR7 inflammatory myeloid cells, CD11b^+^/F4/80^+^/MHCII^+^/CD206^−^ M1‐like macrophages, and CD11b^+^/F4/80^+^/MHCII^−^/CD206^+^ M2‐like macrophages [50, 51, 52, 53, 54, 55, 56]. MDSCs and M2‐like macrophages act as pro‐tumor populations, while inflammatory myeloid cells and M1‐like macrophages are anti‐tumor in nature [50, 51, 52, 53, 54, 55, 56]. We, therefore, tested whether these sub‐populations were present and/or changed in our tumor models. Slit2 treatment did not alter inflammatory myeloid cells (Fig. 5C,D). In contrast, the Slit2 treatment significantly increased the number of M1‐like macrophages (Fig. 5E,F). Interestingly, Slit2 also reduced the number of pro‐tumor M2‐like macrophages (Fig. 5E,F) and MDSCs (Fig. 5G,H). Therapeutically activating macrophages have been shown to efficiently restrict tumor growth [50, 52, 57]. We further extended our analysis to phagocytic macrophages in these tumors. Flow cytometry analysis of macrophages phagocytosed tumor cells revealed that the Slit2‐treated tumors harbor a significantly increased number of phagocytic macrophages (Fig. 5I,J).

### 3.6 Slit2/Robo1 inhibits Tgf‐β1/β‐catenin signaling in SCLC tumor cells

**Fig. 6:**
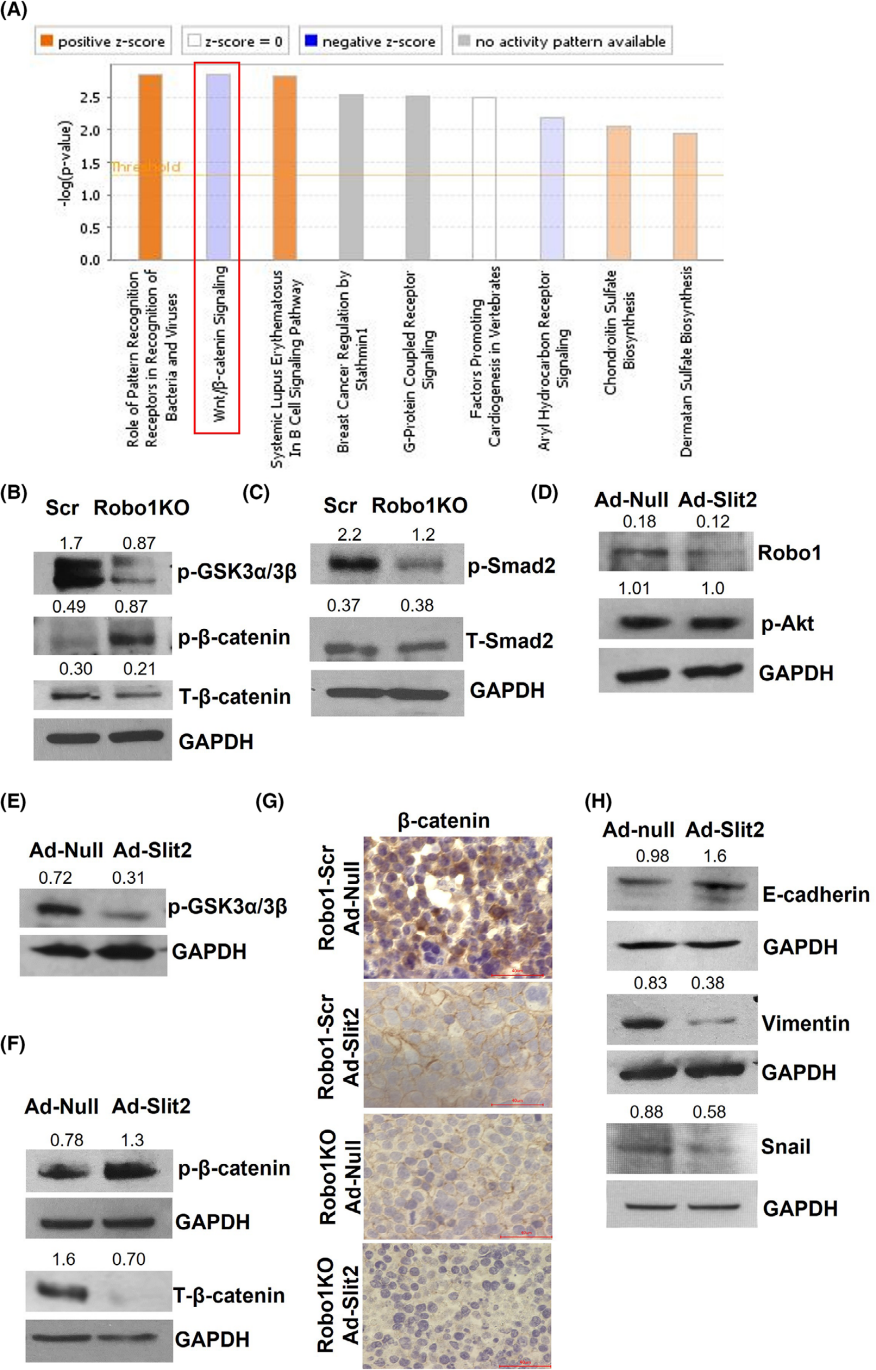
Slit2/Robo1 signaling suppresses Tgf‐β1/GSK3/β‐catenin signaling pathway in tumor cells. (A) Total RNA sequencing data from SBC5‐Scr and SBC5‐Robo1KO cells was subjected to pathway‐based analysis. The graph depicts top biological pathways altered by Robo1 knockout. (B, C) SBC5‐Scr and SBC5‐Robo1KO cell lysates were analyzed for the levels of (B) phospho‐GSK3α/3β (p‐GSK3α/3β), phospho‐β‐catenin at S33/47/T41 position (p‐β‐catenin), total β‐catenin, and (C) phospho‐Smad2 (p‐Smad2), total smad2 (T‐Smad2) by WB. (D–F) cell lysates from SBC5 cells transduced with adenovirus expressing empty vector (ad‐null) or Slit2 (ad‐Slit2) were analyzed for the levels of (D) Robo1, phospho‐AKT (p‐AKT); (E) phospho‐GSK3α/3β (p‐GSK3α/3β); (F) phospho‐β‐catenin at S33/47/T41 position (p‐β‐catenin), total β‐catenin by WB. (G) the representative images of tumor tissue sections showing β‐catenin using IHC method. Scale bar is 40 μm. (H) Cell lysates from SBC5 cells transduced with adenovirus expressing empty vector (ad‐null) or Slit2 (ad‐Slit2) were analyzed for the levels of E‐cadherin, vimentin, and snail using WB. GAPDH was used as a loading control for all the WB analysis. All quantitative data were presented as mean ± SD of three replicates from three independent experiments.

To elucidate the molecular mechanisms used by the Slit2/Robo1 pathway to regulate tumor growth, we performed RNA sequencing on SBC5‐Robo1 KO and SBC5‐Scr cells. Analysis of differentially expressed genes from these samples revealed a suppressed Wnt/β‐catenin signaling pathway in SBC5‐Robo1KO cells (Fig. 6A). These signaling changes were independently evaluated using western blots, which confirmed that β‐catenin is inhibited in SBC5‐Robo1KO cells (Fig. 6B). Crosstalk between Slit2/Robo1 and Tgf‐β1/β‐catenin signaling has previously been identified in heart and breast tissue [58, 59]. Tgf‐β1 and β‐catenin are important oncogenic drivers, and their levels inversely correlate with prognosis in many cancers, including SCLC [60, 61, 62, 63, 64]. However, an interaction between Slit2/Robo1 and Tgf‐β1/β‐catenin signaling in SCLC is untested. Therefore, we analyzed the crosstalk of Slit2/Robo1 and Tgf‐β1 signaling in SCLC. Western blot analysis showed that the activation of Smad2, the downstream signaling molecule of Tgf‐β, was reduced in SBC5‐Robo1KO cells compared with SBC5‐Scr (Fig. 6C). We next evaluated the effect of Slit2 overexpression on cell proliferation pathways. We observed that Slit2 overexpression reduced the levels of Robo1 (Fig. 6D) but did not change the cell proliferation marker, AKT (Fig. 6D). Next, we evaluated the GSK3/β‐catenin signaling pathways [65, 66] and found that Slit2 overexpression reduces the phosphorylation of GSK3 (Fig. 6E) and resulted in increased phosphorylation of β‐catenin and a diminished overall total β‐catenin (Fig. 6F). We also observed reduced levels of β‐catenin in SBC5 Robo1 KO and Slit2‐treated xenograft tumors compared with PBS‐treated SBC5‐Scr tumors (Fig. 6G). Interestingly, β‐catenin in SBC5‐Robo1KO and Slit2‐treated xenografts was translocated to the cell membrane, while it was present in the nucleus of SBC5‐Scr tumor cells (Fig. 6G).

Invasive tumor cells show epithelial‐to‐mesenchymal transition (EMT) characterized by reduced expression of the epithelial marker, E‐cadherin, and increased expression of mesenchymal markers, Vimentin, and Snail [67]. Translocation of β‐catenin to cell membrane stabilizes E‐cadherin to improve cell–cell adhesion and reduce invasiveness [68, 69]. Analysis of EMT markers showed that Slit2 overexpression enhances the expression of E‐cadherin and reduces the expression of Vimentin and Snail (Fig. 6H). These results suggest that Slit2 overexpression and Robo1 knockout suppresses tumorigenic signaling pathways, including EMT and proliferation pathways.

### 3.7 Slit2 inhibits Tgf‐β1/β‐catenin signaling in macrophages

**Fig. 7:**
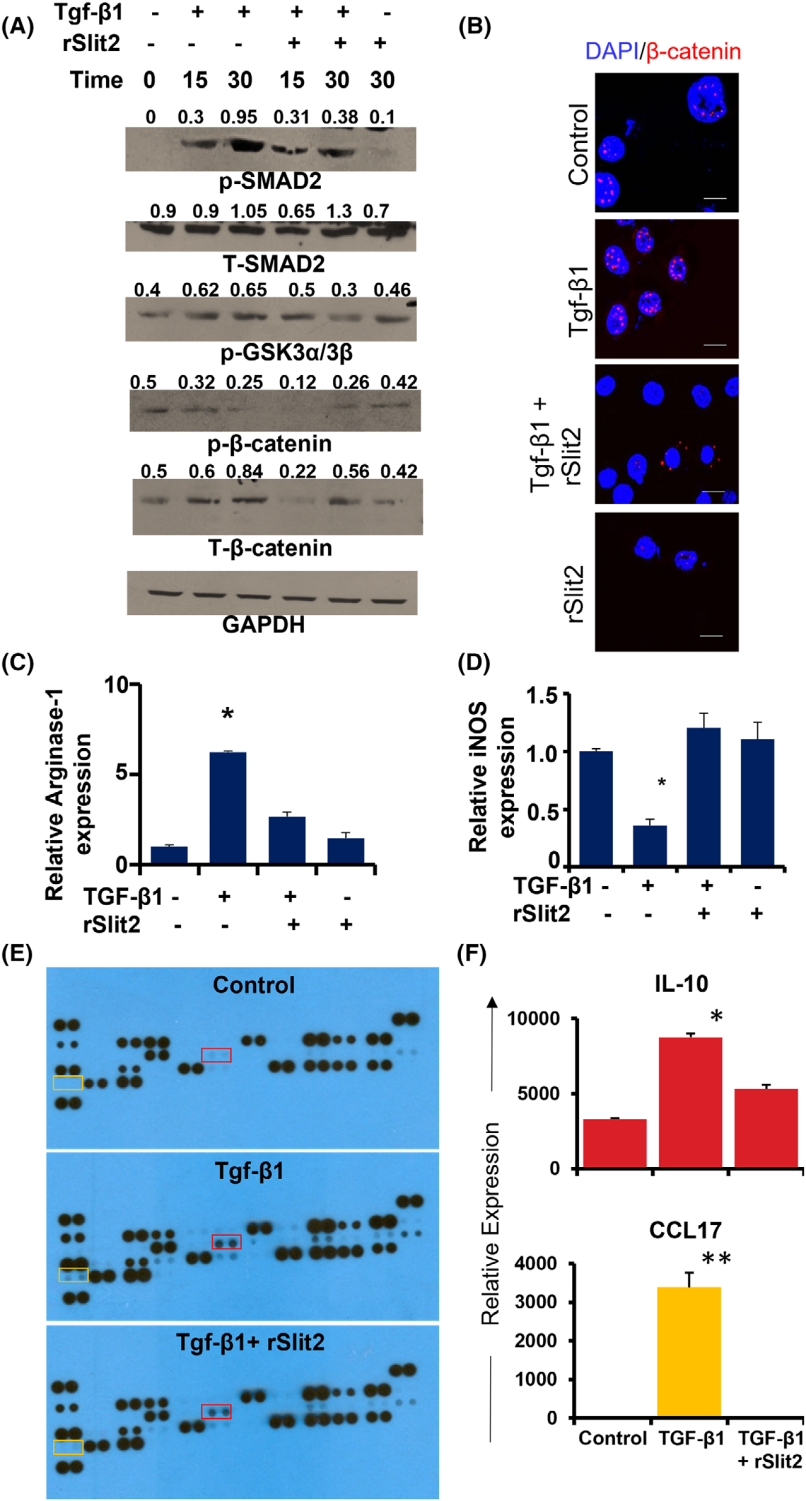
Slit2 inhibits Tgf‐β1/β‐catenin signaling in macrophages. rSlit2 pre‐treated or no‐treatment BMDMs were stimulated with or without Tgf‐β1 and analyzed for the levels of (A) phospho‐Smad2 (p‐Smad2), total smad2 (T‐Smad2), phospho‐GSK3α/3β (p‐GSK3α/3β), phospho‐β‐catenin at S33/47/T41 position (p‐β‐catenin), total β‐catenin by WB; (B) total β‐catenin by immunofluorescence (scale bar is 20 μm); (C) M2 marker arginase 1; (D) M1 marker iNOS by real time PCR; (E) cytokine/chemokine molecules by protein array. (F) Quantification of IL‐10 (red box) and CCL17 (yellow box) detected in (E). All quantitative data were presented as mean ± SD of three replicates from three independent experiments. * is *P* < 0.05, ** is *P* < 0.01 using one way ANOVA test.

Tgf‐β1/β‐catenin signaling can promote M2‐like TAMs and inhibit β‐catenin reprogramming of M2‐like TAMs to M1‐like macrophages [70]. Using primary bone marrow‐derived macrophages (BMDM), we found that Slit2 inhibits Tgf‐β1‐mediated activation of Smad2 (Fig. 7A). We confirmed the expression of the Robo1 receptor on BMDMs by flow cytometry (Fig. S1). Further analysis showed that total GSK3 was increased and that phosphorylated‐GSK3 levels were reduced in Slit2‐treated BMDMs (Fig. 7A). In agreement, we also observed increased phosphorylated β‐catenin and reduced total β‐catenin levels in Slit2‐treated BMDMs (Fig. 7A). To independently test this observation, we measured β‐catenin levels in Tgf‐β1 activated BMDMs by immunofluorescence analysis and found reduced β‐catenin (Fig. 7B). In agreement with our data from SCLC tumor cells, Slit2‐treated BMDMs showed translocation of β‐catenin outside of the nucleus (Fig. 7B). Tgf‐β1 has been shown to activate M2‐like TAMs in the lungs [71]. To evaluate whether Slit2 can antagonize Tgf‐β1 induced M2‐like macrophage polarization, we analyzed the mRNA expression levels of M1‐like marker iNOS and M2‐like marker Arginase‐1. RT‐PCR analysis revealed that Slit2 significantly inhibits the expression of Arginase‐1 (Fig. 7C) and promotes iNOS (Fig. 7D) in the Tgf‐β1‐treated BMDMs. We next evaluated the effect of Slit2 on the cytokine/chemokine profile of Tgf‐β1 activated BMDMs. Protein array analysis identified significant increases in the expression of both CCL17 and IL‐10 following Tgf‐β1 treatment and that these effects could be inhibited by Slit2 pre‐treatment (Fig. 7E,F). These findings suggest that Slit2 inhibits the polarization of macrophages towards an M2‐like phenotype by suppressing the Tgf‐β1/β‐catenin signaling pathway.

## 4 Discussion

Slit2 has tumor‐suppressive activity in several human malignancies, however, its role in SCLC biology is undefined. Here, we show that Slit2 expression levels are reduced in SCLC tumors relative to the adjacent normal lung in three independent tumor cohorts. Recent transcriptomic advances have enabled the stratification of SCLC into four distinct subtypes, each defined by the expression of transcription factors including ASCL1 (SCLC‐A), NEUROD1 (SCLC‐N), POU2F3 (SCLC‐P), and YAP1 (SCLC‐Y) [47]. These subtypes display differing immune infiltrates and patient survival rates, providing a new resource to determine the molecular features contributing to these outcomes. In this work, we find that although Slit2 levels are diminished in SCLC tumors, the SCLC‐Y subtype, which has the best prognosis for SCLC patients [49], has the highest expression of Slit2, compared with more aggressive SCLC. These correlative findings suggest that Slit2 may have an important role in suppressing SCLC tumorigenesis. In this study, we expanded our computational analysis to test the role of Slit2 in SCLC biology. Our work showed that Slit2 does inhibit SCLC tumor growth, cell migration, and local invasion *in vitro* and *in vivo*. These findings are in agreement with data from non‐small‐cell lung cancer (NSCLC), suggesting that Slit2 function is widely important for preventing lung cancer tumorigenesis [72, 73]. In support of this observation, Srivastava *et al*. [74] also reported a tumor‐suppressive activity of Slit2/Robo1 induced signaling in the head and neck and squamous cell carcinoma of the lungs.

Slit2 is an extracellular ligand that can directly bind to the Robo1 cell surface receptor [25, 27], implicating Robo1 as a player in SCLC biology. Little is currently known about the role of Robo1 in SCLC. Our analysis of human samples showed that the Robo1 mRNA levels are elevated in SCLC tumors compared with adjacent normal lung, in contrast to Slit2. This shows that there is an inverse correlation between Slit2 and Robo1 expression in SCLC tumors. In support of this finding, we show that Robo1 levels are significantly reduced by the overexpression of Slit2 in SCLC cells. Robo1 has an important role in promoting SCLC phenotypes, as we show that Robo1^−/−^ SCLC cells have diminished migration, invasion, proliferation, and colony formation abilities. Slit2 tumor‐suppressor activity acts primarily through Robo1, as the treatment of Robo1^−/−^ SCLC with rSlit2 protein, does not further inhibit the tumorigenic properties of these cells. These studies imply that Robo1 may act as an oncogene in the absence of its ligand Slit2. Consistent with this observation, a previous study has shown that Robo1 promotes hepatocellular carcinoma in the absence of its ligand Slit2 [33]. Tumor initiating function of Robo1 is also evident in the development of bronchial epithelial hyperplasia and focal dysplasia in Robo1 knockout mice [75]. Based on these results, we propose that Slit2 directly binds to Robo1 and acts as an important tumor‐suppressor, however, in the absence of Slit2, Robo1 promotes SCLC tumorigenesis in a ligand‐independent manner.

Although Slit2 has been identified as a tumor suppressor in several malignancies, its role in regulating the tumor microenvironment and immune infiltrates is undefined [10, 13, 72, 76]. The SCLC tumors with low neuroendocrine features harbor more immune cells compared with SCLC tumors with high neuroendocrine features [77]. By profiling the immune cells in Slit2‐ and control‐treated SCLC tumors, we found higher numbers of anti‐tumor M1‐like macrophages. In contrast, the number of pro‐tumorigenic MDSCs and M2‐like macrophages was significantly reduced. To determine the mechanisms regulating this processing, we analyzed pathways responsible for macrophage fate and found that Slit2 can suppress Tgf‐β1‐induced polarization of BMDMs towards M2‐like macrophages. We found that the Slit2 treatment inhibited Tgf‐β1 downstream signaling molecules. Our results show increased numbers of phagocytic macrophages in the Slit2‐treated tumors and demonstrate a novel function of Slit2 in activating tumoricidal macrophages in SCLC. Therapeutically activating macrophages have been shown to efficiently restrict tumor growth in different cancers, including SCLC [50, 52, 57].

Several mechanisms are utilized by SCLC for their aggressive properties, however, EMT, is widely used. EMT is characterized by the loss of epithelial characteristics and polarity, as well as the acquisition of mesenchymal phenotype with disrupted cell–cell contact, increased motility, invasiveness, and metastatic behavior [67]. In this work, we found that Slit2 overexpression resulted in decreased Snail and Vimentin levels. In contrast, Slit2 overexpression increased E‐cadherin levels, suppressing EMT. Tgf‐β1 is a master regulator of the EMT in cancer cells and has been linked to EMT phenotype in a plethora of tumor types [78, 79]. In addition, Tgf‐β1 signaling has been shown to Robo1 expression in mammary epithelial cells [59], suggesting that the Slit2 binding of Robo1 may inhibit the EMT transition of SCLC by suppressing Tgf‐β1 signaling.

Wnt‐mediated upregulation of β‐catenin signaling has emerged as a critical oncogenic pathway in lung cancer [80]. We have found that Slit2 overexpression or Robo1 knockout inhibits β‐catenin levels in SCLS cells. Imaging analysis showed that the Slit2 treatment triggers the translocation of β‐catenin from the nucleus to the cell membrane. It has been shown that nuclear β‐catenin activates oncogenic signaling, while membranous β‐catenin stabilizes E‐cadherin to reduce cell invasiveness. [81]. High expression of β‐catenin has been detected in 60% of lung cancer when compared with normal lung tissue [76]. Although Slit2 has been reported to inhibit the growth of different tumors via β‐catenin signaling, little is known about its role in regulating β‐catenin signaling in macrophages [13, 15, 19, 82]. In addition to targeting tumor cell proliferation, our results show that Slit2 activates anti‐tumor macrophages, inducing anti‐tumor TME. We find that Slit2 can suppress Tgf‐β1‐induced polarization of macrophages towards pro‐tumor M2‐like macrophages by suppressing β‐catenin levels [70]. Furthermore, Slit2 reduced the expression of CCL17 and IL‐10 in Tgf‐β1 activated M2 macrophages. M2‐TAMs that secrete CCL17 and IL‐10 have been shown to promote tumor growth [83, 84, 85, 86]. Overall, our data show that Slit2 suppresses the GSK3/β‐catenin signaling pathway in the tumor cells and macrophages to inhibit SCLC growth. In summary, our results for the first time, establish a tumor‐suppressive role of Slit2 and tumor‐promoting role of Robo1 in SCLC.

## 5 Conclusion

Analysis of human samples showed reduced levels of Slit2 and increased levels of Robo1 in SCLC tumors, confirming the tumor‐suppressive function of Slit2 and the oncogenic role of Robo1 observed in our *in vitro* and *in vivo* studies. Our results showing increased Slit2 expression levels in the SCLC subtype linked to improved survival demonstrated the translational significance of Slit2 as a therapeutic agent against SCLC. Overall, these studies indicate Slit2/Robo1 as a novel prognostic and therapeutic strategy to target highly aggressive SCLC that lacks effective therapies.

## Author contributions

RKG was responsible for the conceptualization and design of the project, acquisition of funding, supervision, manuscript review and editing, and project administration. DKA was responsible for conceptualization and designing the project, research protocol development, performing experiments, data collection, analysis, and curation, and writing the original manuscript draft. BP was responsible for performing experiments, collecting, and analyzing data, and writing the original manuscript. MC was responsible for generating reagents and cell lines, performing experiments, collecting data, and manuscript review and editing. S Mishra was responsible for analyzing data and manuscript review and editing. S Misri was responsible for performing experiments, collecting, and analyzing data, and manuscript review and editing. KKW was responsible for manuscript review and editing. SS was responsible for conducting research and manuscript review and editing. WOM, JS, and VSG performed data analysis using publicly available datasets and manuscript review and edit.

## Conflict of interest

The authors declare no conflict of interest.

### Peer review

The peer review history for this article is available at https://publons.com/publon/10.1002/1878‐0261.13289.

## Supporting information


**Fig. S1.** Flow cytometry analysis of Robo1 expression in BMDMs.
**Table S1.** RNA expression levels of SCLC subtype‐specific transcription factors.

## Data Availability

The data that support the findings of this study are available in the main figures and supplementary material of this article.
