# Supplementary material for: Slit2/Robo1 signaling inhibits small‐cell lung cancer by targeting β‐catenin signaling in tumor cells and macrophages
Source: Mol Oncol. 2023 Jan 10;17(5):839–56. doi: 10.1002/1878-0261.13289 (PMC10158774; doi:10.1002/1878-0261.13289)
Supplement: Supplementary file 1 — Fig. S1. Flow cytometry analysis of Robo1 expression in BMDMs. Table S1. RNA expression levels of SCLC subtype‐specific transcription factors. [file MOL2-17-839-s001.zip › MOL2_13289_Suppl. legends.docx]

**Suppl. Fig. 1.** BMDMs were harvested from mice and Robo1 expression was analyzed using the anti-Robo1 antibody by flow cytometry.

**Supplementary Table 1**. The expression of specific transcription factors was analyzed based on their RNA expression across SCLC patients.
