# Supplementary material for: Slit2/Robo1 signaling inhibits small‐cell lung cancer by targeting β‐catenin signaling in tumor cells and macrophages
Source: Mol Oncol. 2023 Jan 10;17(5):839–56. doi: 10.1002/1878-0261.13289 (PMC10158774; doi:10.1002/1878-0261.13289)
Supplement: Supplementary file 1 — Fig. S1. Flow cytometry analysis of Robo1 expression in BMDMs. Table S1. RNA expression levels of SCLC subtype‐specific transcription factors. [file MOL2-17-839-s001.zip › MOL2_13289_Supplementary Information.docx]

**Suppl. Fig. 1.** Flow cytometry analysis of Robo1 expression in BMDMs.

**Supplementary Table 1**. RNA expression levels of SCLC subtype-specific transcription factors.

|  | mRNA expression levels | | | |  |
| --- | --- | --- | --- | --- | --- |
| PATIENT # | ASCL1 | NEUROD1 | POU2F3 | YAP1 | SUBTYPE |
| 1 | 12.6783 | 7.2908 | 3.1396 | 5.89059 | ASCL1 |
| 2 | 4.05605 | 6.12703 | 9.38656 | 6.84723 | POU2F3 |
| 3 | 3.95761 | 6.18488 | 9.04676 | 7.56059 | POU2F4 |
| 4 | 13.0951 | 6.06999 | 3.4114 | 6.0568 | ASCL1 |
| 5 | 4.68577 | 7.89516 | 5.97666 | 5.3458 | NEUROD1 |
| 6 | 12.5527 | 6.29742 | 2.99814 | 6.12335 | ASCL1 |
| 7 | 12.2472 | 6.11777 | 3.50129 | 6.56754 | ASCL1 |
| 8 | 4.00364 | 5.95437 | 3.49817 | 9.17876 | YAP1 |
| 9 | 8.64391 | 6.09002 | 3.35309 | 8.83994 | YAP1 |
| 10 | 12.1674 | 6.64523 | 3.60881 | 5.44005 | ASCL1 |
| 11 | 4.93655 | 6.31292 | 3.4816 | 8.07735 | YAP1 |
| 12 | 5.326 | 6.15745 | 3.76768 | 5.30568 | NEUROD1 |
| 13 | 12.4406 | 6.77994 | 3.4266 | 7.46003 | ASCL1 |
| 14 | 4.19613 | 6.02336 | 10.8213 | 5.69899 | POU2F3 |
| 15 | 7.277 | 6.23625 | 3.46776 | 7.67334 | YAP1 |
| 16 | 5.6209 | 6.26065 | 3.61129 | 4.90264 | NEUROD1 |
| 17 | 12.3886 | 6.38337 | 3.22029 | 7.35978 | ASCL1 |
| 18 | 12.6105 | 6.42705 | 4.20837 | 7.56022 | ASCL1 |
